# Using the Relationship between Concentrations of Selected Whey Proteins and BHBA to Characterize the Metabolism of Dairy Cows in Early Lactation

**DOI:** 10.3390/ani11082298

**Published:** 2021-08-04

**Authors:** Kamila Puppel, Patrycja Staniszewska, Marcin Gołębiewski, Jan Slósarz, Grzegorz Grodkowski, Paweł Solarczyk, Małgorzata Kunowska-Slósarz, Piotr Kostusiak, Beata Kuczyńska, Tomasz Przysucha

**Affiliations:** Institute of Animal Science, Warsaw University of Life Sciences, 02-786 Warsaw, Poland; patrycja_staniszewska@sggw.edu.pl (P.S.); jan_slosarz@sggw.edu.pl (J.S.); grzegorz_grodkowski@sggw.edu.pl (G.G.); pawel_solarczyk@sggw.edu.pl (P.S.); malgorzata_kunowska_slosarz@sggw.edu.pl (M.K.-S.); piotr_kostusiak@sggw.edu.pl (P.K.); beata_kuczynska@sggw.edu.pl (B.K.); tomasz_przysucha@sggw.edu.pl (T.P.)

**Keywords:** metabolic disease, BHBA, α-lactalbumin, β-lactoglobulin, dairy cow

## Abstract

**Simple Summary:**

A negative energy balance alters the concentration of various fractions of cows’ milk. Moreover, key changes are observed in the fat and protein content. Therefore, by analyzing the concentration of individual proteins in milk, it is possible to indirectly monitor the health of the animal. Subclinical ruminal acidosis and ketosis are the primary economic issues in dairy farming due to non-specific symptoms, difficulty in obtaining a diagnosis and reduced milk production. The aim of the present study was to identify the relationship between the concentrations of blood β-hydroxybutyric (BHBA) and whey proteins in milk as a marker for the diagnosis of metabolic diseases. Whey proteins were significantly influenced by both the lactation phase and BHBA. Therefore, it can be concluded that whey proteins can be used as non-invasive markers for diagnosing metabolic diseases. A high concentration of β-lactoglobulin can be a marker for diagnosing ketosis. Conversely, elevated levels of α-lactalbumin may indicate the occurrence of a metabolic disorder, such as acidosis.

**Abstract:**

A negative energy balance alters the concentration of various fractions of cows’ milk. Therefore, by analyzing the concentration of individual proteins in milk, it is possible to indirectly monitor the health of the animal. The aim of the present study was to identify the relationship between the concentrations of blood β-hydroxybutyric acid (BHBA) and whey proteins in milk as a marker for the diagnosis of metabolic diseases. The analysis included milk and blood samples from 95 Holstein-Friesian cows, which were divided into three groups that were differentiated in terms of serum BHBA levels 5–7 days post-calving: LBHBA, low level of BHBA: 0.200–0.500 mmol/L; NBHBA, optimal level of BHBA- control group: 0.500–1.200 mmol/L; HBHBA, high level of BHBA: >1.200 mmol/L. Concentrations of α-lactoalbumin in the milk after 7 days of lactation proceeded in accordance with the concentration of β-hydroxybutyric acid, as follows: LBHBA > NBHBA > HBHBA. Concentrations of β-lactoglobulin in milk after 14 days of lactation proceeded in accordance with the concentration of β-hydroxybutyric acid, as follows: LBHBA < NBHBA < HBHBA. Therefore, it can be concluded that whey proteins can be used as non-invasive markers for diagnosing metabolic diseases. A high concentration of β-lactoglobulin can be a marker for diagnosing ketosis. Conversely, elevated levels of α-lactalbumin may indicate the occurrence of a metabolic disorder, such as acidosis.

## 1. Introduction

During the past 50 years, there has been significant progress in the performance of dairy cows, and a yield of greater than 9,000 kg of milk per lactation is easily achieved [1]. However, the breeding work that has been conducted has adversely affected the health of animals and the quality of the milk. A serious issue on dairy farms is the occurrence of metabolic diseases, such as ketosis, subclinical and clinical acidosis, or abomasal dislocation [1,2]. A negative energy balance (NEB) is partly caused by cows limiting their intake of dry matter during the postpartum period and is the most common cause of metabolic diseases. The primary method of diagnosing ketosis, in addition to clinical symptoms, is the determination of biochemical blood indicators [3,4]. During the course of the disease, there is an increase in ketone compounds and free fatty acids and a decrease in cholesterol and glucose levels [5]. The most used parameter for detecting ketosis is the concentration of β-hydroxybutyric acid (BHBA) in blood. A concentration of BHBA greater than 1.2 mmol/L and less than 2.6 mmol/L indicates the presence of subclinical ketosis [1,6]. Animals with concentrations of BHBA exceeding 2.6 mmol/L show signs of clinical ketosis, although individual variations concerning the tolerance of this indicator are large [7]. In general, just before calving, the BHBA concentration does not exceed 0.575–0.750 mmol/L (in the absence of a negative energy balance and no ketogenic silage). Shortly after calving, the BHBA concentration may increase rapidly, and its increase to a range of 1.0–1.2 mmol/L indicates a high risk of perinatal disease [1,3,6,7].

Acidosis is a significant metabolic disorder that occurs among dairy cows, primarily in early lactation. A distinction is made between a subclinical form, referred to as subclinical ruminal acidosis or subacute ruminal acidosis (SARA), and clinical acidosis (acute acidosis) [8,9,10]. During the development of acidosis, large amounts of lactic acid and toxic amines (histamine and tyramine) are formed in the rumen. Leighton et al. [11] reported that the process of ketogenesis occurs mainly in the rumen epithelium (RE), as more ketone bodies are synthesized in the RE compared to the liver in the fed state. Short-chain fatty acids can be converted to 3-hydroxy-3-methylglutaryl-coenzyme A by HMG-CoA synthase in the mitochondria, which proceeds to ketogenesis [12]. Additionally, Owens et al. [13] reported that when cattle are transitioned abruptly to a high-grain diet, such as during the periparturient period, the rate of short-chain fatty acid production often exceeds the rate of buffering and absorption through the RE, making the cow more susceptible to developing the digestive disorder known as ruminal acidosis. The concept of using the milk fat to protein ratio (T/P) to diagnose SARA is based on the assumption that rumen fermentation diseases caused by acidosis result in reduced milk fat content (the effect on reduced protein content is much smaller). Unfortunately, diagnosing SARA based on the ratio of fat to protein in milk is not reliable. According to Enemark [14], a negative correlation (r = −0.06) was observed between the rumen fluid pH and milk fat content in cows during the first 30 days of lactation, suggesting that this indicator is not useful. Subclinical ruminal acidosis and ketosis are the primary economic issues in dairy farming due to non-specific symptoms, difficulty in obtaining a diagnosis and reduced milk production. A metabolic disease alters the concentration of various fractions of cows’ milk. Moreover, key changes are observed both in fat and protein content. However, the research conducted to date has not considered the relationship between the occurrence of ketosis and acidosis and the development of the content of selected whey proteins (α-lactalbumin, α-LA; β-lactoglobulin, β-LG) in milk. The hypothesis assumes that the concentrations of α-LA and β-LG could characterize the metabolism of dairy cows in early lactation. Therefore, the aim of this study was to identify the relationship between the concentrations of blood β-hydroxybutyric acid and α-lactalbumin and β-lactoglobulin in milk as a tool for the diagnosis of metabolic diseases.

## 2. Materials and Methods

### 2.1. Animals and Sampling

During the experiment, the herd was under veterinary control. The study was conducted at the experimental dairy farm of the Warsaw University of Life Sciences (WULS) on a herd of approximately 350 cows that were maintained in a free-stall housing system. The average performance of the cows exceeded 11,000 kg of milk per lactation, with 3.42% protein and 4.38% fat content. The analysis included milk and blood samples from 95 Holstein-Friesian cows (multiparous, in the second lactation, an average body weight of 691 ± 28 kg), which were divided into three groups that were differentiated in terms of clinical symptoms (Table 1) and serum BHBA levels 5–7 days post-calving: 30 acidotic cows whose serum BHBA concentration was 0.200–0.500 mmol/L- LBHBA, 37 healthy cows whose serum BHBA concentration was 0.51–1.2 mmol/L- NBHBA, and 28 ketotic cows whose serum BHBA concentration was > 1.2 mmol/L- HBHBA.

The cows’ feeding regime was based on the total mixed ration (TMR) diet (ad libitum). Ingredient composition of the TMR (kg/d DM) was as follows: maize silage—10.05; alfalfa silage—3.80; corn silage—2.41; soybean meal—2.57; pasture ground chalk—0.20; salt—0.05; rapeseed meal—2.12; magnesium oxide 0.07. Chemical composition of the TMR (g/kg DM) was as follows: ash—5.27; crude protein—16.10; fat—4.92; starch—291.74; sugar—76.21; acid detergent fiber—31.11; neutral detergent fiber—42.04. The remaining factors characterizing TMR were as follows: total kg of DM—22.21; daily intake (kg/d DM)—20.01; *netto energy lactation* (Mcal/kg)—1.74; average milk production (kg/d)—36.21; unit of milk production balance (%)—3.41; protein digested in the small intestine when rumen-fermentable nitrogen is limiting balance (%)—2.61; protein digested in the small intestine when rumen-fermentable energy is limiting balance (%)—2.33.

Samples of milk and blood were collected from the cows for laboratory analyses at weekly intervals. The samples were obtained at six time points: between days in milk 5–7, 8–14, 15–21, 22–28, 29–35 and 36–42, resulting in 570 samples of both milk and blood. The milk samples (250 mL) were obtained from each cow (during an evening and morning milking to ensure a representative sample is collected and then pooled) using milk samplers to sterile bottles, preserved with Mlekostat CC and immediately transported to WULS for compositional analysis. The blood samples (10 mL) were obtained from each cow immediately after morning milking via jugular vein puncture using a tube (Vacuette, Germany) containing potassium-EDTA (K3EDTA, 1.8 g/L of blood) as an anticoagulant. Subsequently, they were centrifuged (at 1800× g at 4 °C for 15 min) and transported to the veterinary center at WULS for the analysis of blood plasma metabolites (BHBA).

### 2.2. Chemical Analyses

The basic parameters of milk, i.e., fat, protein, lactose and casein content, were determined via automated infrared analysis using a Milkoscan FT 120 analyzer (Foss Electric, Hillerød, Denmark).

Concentrations of α-lactoalbumin and β-lactoglobulin were determined using an Agilent 1100 Series RP-HPLC (Agilent Technologies, Waldbronn, Germany) according to the methodology described by Puppel et al. [6]. All samples were analyzed in duplicate. The identification of peaks as α-lactalbumin and β-lactoglobulin was confirmed by comparison with the standards (Sigma-Aldrich, St. Louis, MO, USA).

The level of AspAT, Glucose, BHBA and GGTP was determined using a BS800M biochemical analyzer (PZ Cormay, Warsaw, Poland).

### 2.3. Statistical Analysis

The data were compiled statistically via a multi-factor analysis of variance (ANOVA) by the least-squares method using the IBM SPSS 23.0 package [15]. The distribution of the milk chemical composition and selected whey proteins were examined using the Shapiro–Wilk test. The ANOVA analysis was used to establish the influence of the lactation phase on the chemical composition of the milk and the level of selected whey proteins.

After preliminary analysis of the samples, the cows were divided according to the concentrations of β-hydroxybutyric acid: LBHBA,0.200–0.500 mmol/L (30 cows); NBHBA, 0.500–1200 mmol/L (37 cows); HBHBA, > 1.200 mmol/L (28 cows).

The following statistical model was used:

Y = μ + A_i_ + B_j_ + (A × B)_ij_+ e_ijk_ (1)

where μ—mean, A_i_—day in lactation, B_j_—BHBA concentration, A × B—interaction between day in lactation and BHBA concentration and e_ij_—random error.

Data were presented as least squares means (LSM) with the standard error of the mean (SEM).

## 3. Results and Discussion

During lactation, the energy and protein requirement increases dramatically. In dairy cows, there is a requirement for greater than a five-fold increase in energy and protein from late gestation to lactation [16]. The role of insulin in increasing the milk protein content is considered problematic in dairy cows due to a drastic decrease in the concentration of this hormone in the blood at the beginning of lactation. It has been demonstrated that insulin plays a key role in protein synthesis in dairy cows [17]. Studies have shown that the concentrations of glucose were influenced by BHBA (Table 1). HBHBA was associated with the lowest concentrations of glucose (60% lower than NBHBA and 49% lower than LBHBA). The hallmark of cows diagnosed with ketosis is low insulin levels due to chronic hypoglycemia (low blood glucose) [18], which is confirmed by the obtained results.

Table 2 shows changes in gross milk composition in the first 42 days of lactation. After 14 days of lactation, the concentrations of casein and protein in milk proceeded in accordance with the concentration of β-hydroxybutyric acid, as follows: LBHBA > NBHBA > HBHBA. Gross milk composition was significantly (*p* ≤ 0.01) influenced by both the lactation phase and BHBA. LBHBA was associated with the highest concentrations of casein (13% higher than NBHBA and 24% higher than HBHBA) and protein (13% higher than NBHBA and 29% higher than HBHBA) after the first 14 days of lactation (Table 2).

In a study conducted by Duffield et al. [19], cows with diagnosed ketosis and high concentrations of BHBA had lower percentages of protein in the milk. Additionally, Grieve et al. [20] reported that a decrease in protein concentration was due to a reduced energy supply, which corroborates the results obtained in the present study.

It has been suggested that the adipose tissue of cows diagnosed with ketosis is more susceptible to signals that initiate lipolysis (activation of fat reserves) and, in addition, fat cows exhibit insulin resistance. Regarding insulin resistance, pancreatic cells produce more insulin because tissues do not respond to it and do not absorb glucose. Excess insulin inhibits the enzymes responsible for gluconeogenesis in the liver (the process of forming glucose from the non-sugar compounds propionic acid, lactate acid, glycerol and free amino acids (alanine, glutamic acid)) [18]. In ruminants, BHBA is a precursor of milk fat and supplies approximately half of the de novo synthesized fatty acids [21]. Moreover, butyric acid is absorbed by the RE in ruminants and metabolized to BHBA in the absence of NEB [22]. However, lipid mobilization leads to hyperketonemia, which is characterized by high levels of BHBA in the blood. Concentrations of fat in the milk after 7 days of lactation proceeded in accordance with the concentration of β-hydroxybutyric acid as follows: LBHBA < NBHBA < HBHBA. Studies have shown that the concentration of fat was significantly (*p* ≤ 0.01) influenced by BHBA. HBHBA was associated with the highest concentrations of fat (64% higher than LBHBA and 29% higher than NBHBA). Additionally, a strong correlation (0,300; *p* ≤ 0.01) was found between BHBA and fat (Table 2). Zhang et al. [21] reported that high concentrations of BHBA could increase the synthesis of triglyceride in mammary epithelial cells, which explains an increase in the milk fat in dairy cows with ketosis.

The synthesis of proteins requires the constituents of the protein synthesis machinery, as well as the availability of amino acids and a large supply of energy [23]. Concentrations of BHBA, nonesterified fatty acid, albumin, creatinine, aspartate aminotransferase and alkaline phosphatase can be assessed in blood to indicate energy and protein metabolism and liver health [24]. Proteins, such as casein, α-LA and β-LG, are synthesized from the primary blood constituents in the epithelial cells of the mammary gland [25]. Barbana et al. [26] reported that α-LA could bind to FA. Consequently, in a complex mixture, such as whey, the FA will exist in a complex equilibrium between the total FA/protein complexes and its free form. During the first- and second-week post-calving, an elevated serum BHBA results in a greater milk fat percentage and a lower milk protein percentage [6]. Nasrollahi et al. [27] reported that cows with a low ruminal pH also had a greater albumin concentration (*p* = 0.02) in the blood compared with cows with a high and medium ruminal pH (3.91 vs. 3.81 and 3.75 g/dL). A strong negative correlation (−0.310; *p* ≤ 0.01) was found between BHBA and protein (Table 2). The results indicated that cows that differed in BHBA also had various concentrations of protein in milk.

**Figure 1:**
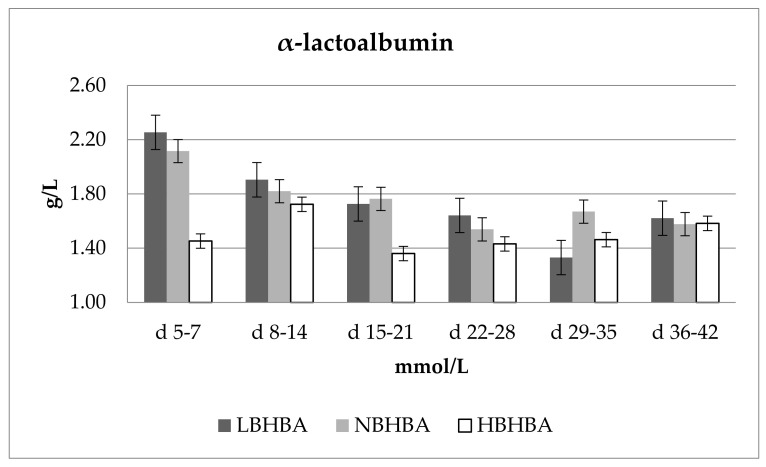
Changes in the concentration of α-lactoalbumin for varying concentrations of BHBA. Concentrations of β-hydroxybutyric acid: LBHBA, 0.200–0.500 mmol/L; NBHBA, 0.500–1.200 mmol/L; HBHBA, >1.200 mmol/L. Data were presented as least squares means with standard error of the mean. Statistical differences between BHBA groups at *p* ≤ 0.01.

Figure 1 shows changes in the concentration of α-lactoalbumin for varying concentrations of BHBA. Concentrations of α-lactoalbumin in the milk after 7 days of lactation proceeded in accordance with the concentration of β-hydroxybutyric acid as follows: LBHBA > NBHBA > HBHBA. α-lactoalbumin was significantly (*p* ≤ 0.01) influenced by both the lactation phase and BHBA. LBHBA was associated with the highest concentrations of α-lactoalbumin after the first 7 days of lactation (6% higher than NBHBA and 35% higher than HBHBA).

**Figure 2:**
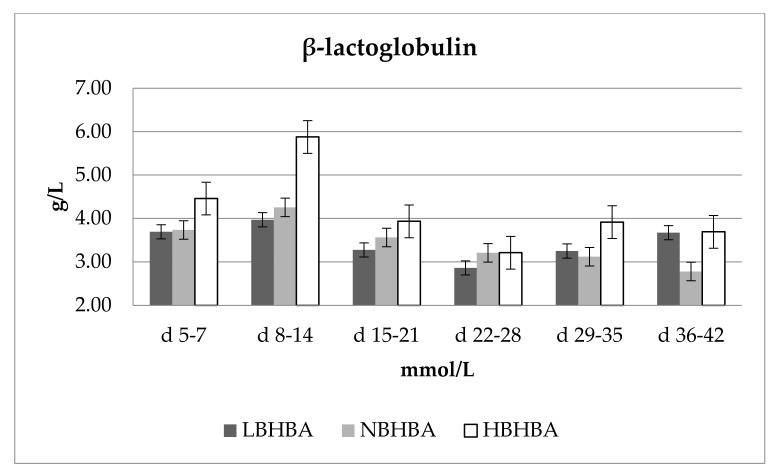
Changes in the concentration of β-lactoglobulin for varying concentrations of BHBA. Concentration of β-hydroxybutyric acid: LBHBA, 0.200–0.500 mmol/L; NBHBA, 0.500–1.200 mmol/L; HBHBA, >1.200 mmol/L. Data were presented as least squares means with standard error of the mean. Statistical differences between BHBA groups at *p* ≤ 0.01.

Puppel et al. [6] reported in the first week of lactation a 34% lower level of conjugated linoleic acid in a group of cows with BHBA levels greater than 1.2 mmol/L compared with cows with BHBA levels lowered than 1.2 mmol/L. Perez et al. [28] and Considine et al. [29] demonstrated that bovine β-LG binds FA in milk, such as myristic acid and conjugated linoleic acid, at a molar ratio of one mole of FA per mole of β-LG dimer. Thus, there is a clear relationship between FA level development and β-lactoglobulin. Figure 2 shows changes in the concentration of β-lactoglobulin for varying concentrations of BHBA. Concentrations of β-lactoglobulin in milk after 14 days of lactation proceeded in accordance with the concentration of β-hydroxybutyric acid as follows: LBHBA < NBHBA < HBHBA. β-lactoglobulin was significantly (*p* ≤ 0.01) influenced by both the lactation phase and BHBA. HBHBA was associated with the highest concentrations of β-lactoglobulin after 14 days of lactation (38.1% higher than NBHBA and 58.4% higher than LBHBA).

*Pearson correlation* analysis showed a significant correlation between BHBA and whey proteins in milk (Table 3). The α-lactalbumin content in milk was inversely correlated with the blood BHBA concentration (−0.695; *p* ≤ 0.01). Moreover, a decrease in α-LA content was observed with an increase in BHBA concentration. The opposite conclusion applied to the β-lactoglobulin content of the milk. A strong correlation was found between BHBA and β-LG (0.757; *p* ≤ 0.01). With an increase in the concentration of BHBA in the blood, the content of a given whey protein in the milk increased.

## 4. Conclusions

In conclusion, the results of this study demonstrate that whey proteins can be used as non-invasive markers for diagnosing metabolic diseases in cows. The results indicated that cows that differed in BHBA also had various concentrations of selected whey proteins in milk. A high concentration of β-lactoglobulin can be a marker for diagnosing ketosis. Conversely, elevated levels of α-lactalbumin may indicate the occurrence of a metabolic disorder, such as acidosis.

## Figures and Tables

**Table 1:** Characteristics of the acidotic, healthy and ketotic cows.

	Acidosis (*n =* 30)		Healthy (*n =* 37)		Ketosis (*n =* 28)	
	LSM	SEM	LSM	SEM	LSM	SEM
Milk Yield (kg/d)	31.221	1.005	29.927	1.024	27.115	0.902
Glucose (mg/dL)	58.223	0.945	63.025	0.887	39.011	0.862
GGTP (U/L)	27.255	0.521	25.027	0.668	72.036	0.754
AspAT (U/L)	127.215	1.114	63.061	0.847	64.052	0.825
BHBA (mmol/L)	0.200–0.500	0.012	0.510–1.200	0.014	> 1.200	0.012
F/P	1.058	0.071	1.212	0.065	1.625	0.045

β-hydroxybutyric acid, BHBA; Gamma-glutamyltranspeptidase, GGTP; Aspartate aminotransferase, AspAT; Fat/Protein ratio, F/P.

**Table 2:** Changes in the basic composition of milk for varying concentrations of BHBA.

Days in Milk	BHBA Group	Casein (%)		Protein (%)		Fat (%)		Lactose (%)		Interaction DIM × BHBA
		LSM	SE	LSM	SE	LSM	SE	LSM	SE	
d 5–7	LBHBA	3.54	0.152	4.51	0.196	4.26	0.687	3.93	0.186	
	NBHBA	3.07	0.058	3.83	0.074	4.84	0.26	4.48	0.07	*p* ≤ 0.01
	HBHBA	2.69	0.076	3.18	0.098	6.6	0.344	4.35	0.093	
d 8–14	LBHBA	2.85	0.068	3.52	0.088	3.13	0.307	4.64	0.083	
	NBHBA	2.96	0.037	3.61	0.047	4.64	0.167	4.74	0.045	*p* ≤ 0.01
	HBHBA	2.79	0.068	3.42	0.088	5.39	0.307	4.6	0.083	
d 15–21	LBHBA	2.72	0.051	3.32	0.065	3.25	0.229	4.79	0.062	*p* ≤ 0.01
	NBHBA	2.75	0.037	3.28	0.047	4.34	0.167	4.9	0.045	
	HBHBA	2.57	0.076	3.02	0.098	5.59	0.344	4.78	0.093	
d 22–28	LBHBA	2.67	0.068	3.11	0.088	3.22	0.307	5.01	0.083	
	NBHBA	2.55	0.048	2.98	0.062	4.7	0.217	4.93	0.059	*p* ≤ 0.01
	HBHBA	2.58	0.108	2.94	0.138	5.48	0.486	4.99	0.131	
d 29–35	LBHBA	2.48	0.088	2.95	0.113	3.76	0.397	4.92	0.107	
	NBHBA	2.55	0.031	3.02	0.04	3.74	0.14	4.96	0.038	*p* ≤ 0.01
	HBHBA	2.3	0.051	2.73	0.065	4.87	0.229	4.73	0.062	
d 36–42	LBHBA	2.65	0.108	3.15	0.138	4.23	0.486	4.87	0.131	
	NBHBA	2.56	0.032	3.07	0.041	3.8	0.143	4.83	0.039	*p* ≤ 0.01
	HBHBA	2.44	0.108	2.9	0.138	4.23	0.486	4.85	0.131	

SE, standard error of the mean; LSM, least-square of the mean; DIM, days in milk. Concentrations of β-hydroxybutyric acid: LBHBA, 0.200–0.500 mmol/L; NBHBA, 0.500–1.200 mmol/L; HBHBA, > 1.200 mmol/L. Statistical differences between BHBA groups at *p* ≤ 0.01.

**Table 3:** Pearson correlations.

	Fat	Protein	Lactose	BHBA	α-LA	β-LG
Fat	1	0.220 **	−0.198 **	0.300 **	−0,136	0.241 **
Protein	0.220 **	1	−0.495 **	−0.310 **	0.178 *	−0.211 **
Lactose	−0.198 **	−0.495 **	1	0.219 **	−0.233 **	0.178 *
BHBA	0.300 **	−0.310 **	0.219 **	1	−0.695 **	0.757 **
α-LA	−0,136	0.178 *	−0.233 **	−0.695 **	1	−0.615 **
β-LG	0.241 **	−0.211 **	0.178 *	0.757 **	−0.615 **	1

** Correlation significant at a level of 0.01. * Correlation significant at a level of 0.05.

## Data Availability

All data generated or analyzed during the study are included in this published article. The datasets used and/or analyzed in the current study are available from the corresponding author on reasonable request.

## References

[1] Puppel K., Kuczyńska B. (2016). Metabolic profiles of cow’s blood; a review. J. Sci. Food Agric..

[2] Gołębiewski M. (2017). Badanie Przydatności Zmodyfikowanej Oceny Kondycji Krów Mlecznych w Zarządzaniu Ich Stadem ze Szczególnym Uwzględnieniem Parametrów Produkcyjnych, Funkcjonowania Układu Rozrodczego Oraz Zdrowia Zwierząt.

[3] Oetzel G.R. (2021). Herd-level ketosis–diagnosis and risk factors. Proceedings of the 40th Annual Conference of Bovine Practitioners.

[4] van der Drift S.G.A., Houweling M., Schonewille J.T., Tielens A.G.M., Jorritsma R. (2012). Protein and fat mobilization and associations with serum beta-hydroxybutyrate concentrations in dairy cows. J. Dairy Sci..

[5] Kanikarla-Marie P., Jain S.K. (2016). Hyperketonemia and ketosis increase the risk of complications in type 1 diabetes. Free Radic. Biol. Med..

[6] Puppel K., Gołębiewski M., Solarczyk P., Grodkowski G., Slósarz J., Kunowska-Slósarz M., Balcerak M., Przysucha T., Kalińska A., Kuczyńska B. (2019). The relationship between plasma β-hydroxybutyric acid and conjugated linoleic acid in milk as a biomarker for early diagnosis of ketosis in postpartum Polish Holstein-Friesian cows. BMC Vet. Res..

[7] Ospina P.A., Nydam D.V., Stokol T., Overton T.R. (2010). Associations of elevated nonesterified fatty acids and beta-hydroxybutyrate concentrations with early lactation reproductive performance and milk production in transition dairy cattle in the northeastern United States. J. Dairy Sci..

[8] Krause K., Oetzel G. (2006). Understanding and preventing subacute ruminal acidosis in dairy herds: A review. Anim. Feed Sci. Tech..

[9] Penner G., Oba M., Gäbel G., Aschenbach J. (2010). A single mild episode of subacute ruminal acidosis does not affect ruminal barrier function in short term. J. Dairy Sci..

[10] Castillo-Lopez E., Wiese B.I., Hendrick S., McKinnon J.J., McAllister T.A., Beauchemin K.A., Penner G.B. (2014). Incidence, prevalence, severity and risk factors for ruminal acidosis in feedlot steers during back grounding diet transition, and finishing. J. Anim. Sci..

[11] Leighton B., Nicholas A.R., Pogson C.I. (1983). The pathway of ketogenesis in rumen epithelium of the sheep. Biochem. J..

[12] Meertens L.M., Miyata K.S., Cechetto J.D., Rachubinski R.A., Capone J.P. (1998). A mitochondrial ketogenic enzyme regulates its gene expression by association with the nuclear hormone receptor PPARα. EMBO J..

[13] Owens F.N., Secrist D.S., Hill W.J., Gill D. (1998). Acidosis in cattle: A review. J. Anim. Sci..

[14] Enemark J.M. (2008). The monitoring, prevention and treatment of sub-acute ruminal acidosis (SARA): A review. Vet. J..

[15] (2021). IBM Crop: Released IBM SPSS for Windows.

[16] Bionaz M., Periasamy K., Rodriguez-Zas S.L., Everts R.E., Lewin H.A., Hurley W.L., Loor J.J. (2012). Old and new stories: Revelations from functional analysis of the bovine mammary transcriptome during the lactation cycle. PLoS ONE.

[17] Griinari J.M., McGuire M.A., Dwyer D.A., Bauman D.E., Barbano D.M., House W.A. (1997). The role of insulin in the regulation of milk protein synthesis in dairy cows. J. Dairy Sci..

[18] Underwood W.J., Blauwiekel R., Delano M.L., Gillesby R., Mischler S.A., Schoell A., James G.F., Lynn C.A., Glen M.O., Kathleen R., Pritchett-Corning M.T.W. (2015). Chapter 15—Biology and Diseases of Ruminants (Sheep, Goats, and Cattle). American College of Laboratory Animal Medicine, Laboratory Animal Medicine.

[19] Duffield T.F., Lissemore K.D., McBride B.W., Leslie K.E. (2009). Impact of hyperketonemia in early lactation dairy cows on health and production. J. Dairy Sci..

[20] Grieve D.G., Korver S., Rijpkema Y.S., Hof G. (1986). Relationship between milk composition and some nutritional parameters in early lactation. Livest. Prod..

[21] Zhang M., Zhang S., Hui Q., Lei L., Du X., Gao W., Zhang R., Liu G., Li X. (2015). β-Hydroxybutyrate Facilitates Fatty Acids Synthesis Mediated by Sterol Regulatory Element-Binding Protein1 in Bovine Mammary Epithelial Cells. Cell Physiol. Biochem..

[22] Månsson H.L. (2008). Fatty acids in bovine milk fat. Food Nutr. Res..

[23] Schimmel P., Alexander R.W., Robert A.M. (2003). Protein Synthesis. Encyclopedia of Physical Science and Technology.

[24] Cozzi G., Ravarotto L., Gottardo F., Stefani A.L., Contiero B., Moro L., Brscic M., Dalvit P. (2011). Short communication: Reference values for blood parameters in Holstein dairy cows: Effects of parity, stage of lactation, and season of production. J. Dairy Sci..

[25] Gellrich K., Meyer H.H.D., Wiedemann S. (2014). Composition of major proteins in cow milk differing in mean protein concentration during the first 155 days of lactation and the influence of season as well as short-term restricted feeding in early and mid-lactation. Czech J. Anim. Sci..

[26] Barbana C., Sanchez L., Perez M.D. (2011). Bioactivity of alpha-lactalbumin related to its interaction with fatty acids: A review. Crit. Rev. Food Sci. Nutr..

[27] Nasrollahi S.M., Zali A., Ghorbani G.R., Kahyani A., Beauchemin K.A. (2019). Short communication: Blood metabolites, body reserves, and feed efficiency of high-producing dairy cows that varied in ruminal pH when fed a high-concentrate diet. J. Dairy Sci..

[28] Perez M.D., Calvo M. (1995). Interaction of beta-lactoglobulin with retinol and fatty acids and its role as a possible biological function for this protein: A review. J. Dairy Sci..

[29] Considine T., Patel H.A., Singh H., Creamer L.K. (2007). Influence of binding conjugated linoleic acid and myristic acid on the heat-and high-pressure-induced unfolding and aggregation of beta-lactoglobulin B. Food Chem..

